# Supplementary material for: CO2 signaling mediates neurovascular coupling in the cerebral cortex
Source: Nat Commun. 2022 Apr 19;13:2125. doi: 10.1038/s41467-022-29622-9 (PMC9019094; doi:10.1038/s41467-022-29622-9)
Supplement: Supplementary file 1 — Supplementary Information [file 41467_2022_29622_MOESM1_ESM.pdf]

Supplementary Information

CO_2 signaling mediates neurovascular coupling in the cerebral cortex

Hosford et al.

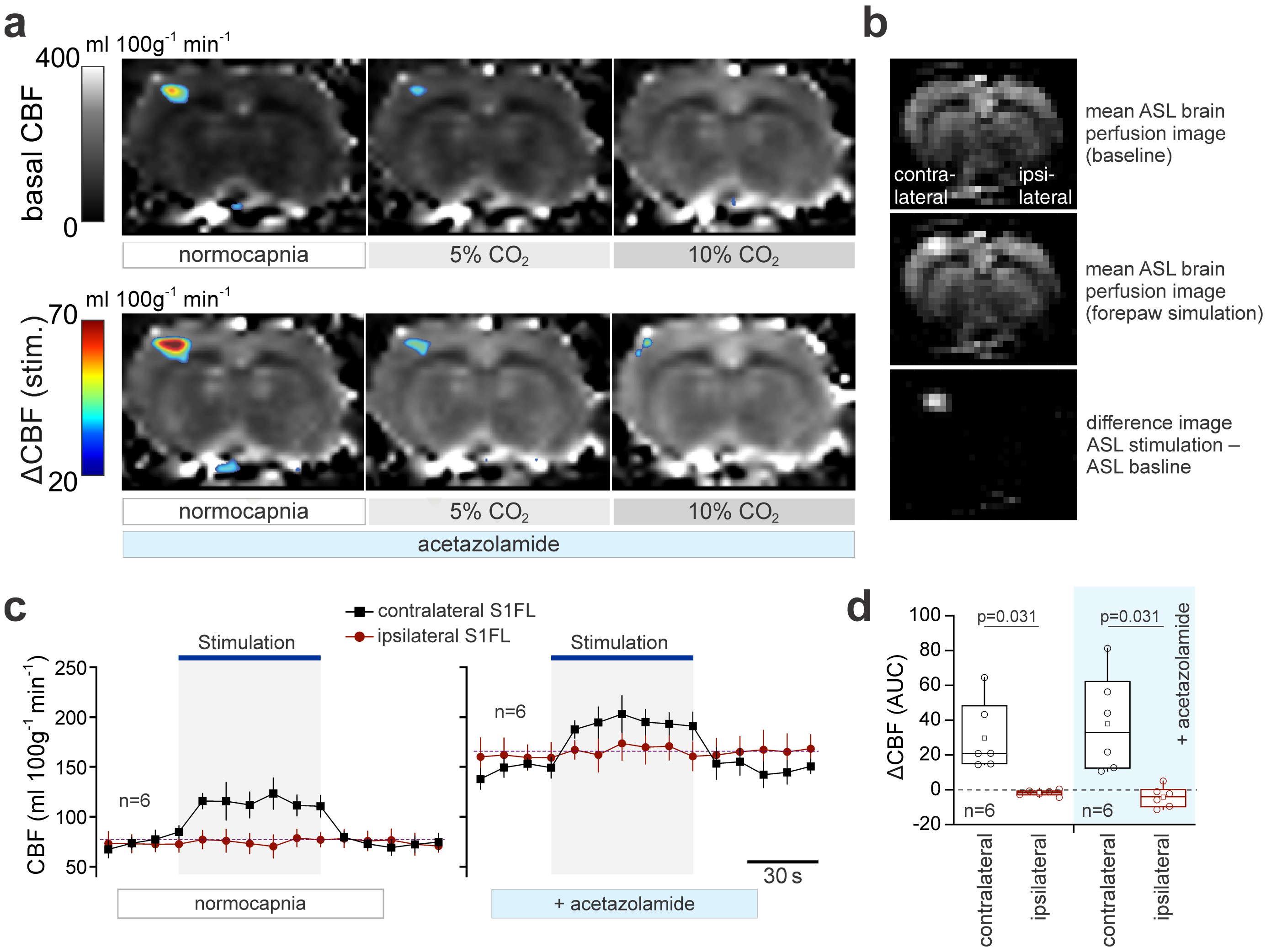

Supplementary Figure 1| **a**, Representative arterial spin labelling (ASL) images of the rat brain illustrating cerebral blood flow (CBF) at baseline (normocapnia), in conditions of 5% and 10% inspired CO_2, after the administration of carbonic anhydrase inhibitor acetazolamide (10 mg kg^{-1}, i.v.), and in conditions of 5% and 10% inspired CO_2, applied concomitantly with systemic carbonic anhydrase inhibition with acetazolamide. Overlaid (false colour scale) illustrates CBF response in the S1FL region of the somatosensory cortex induced by electrical forepaw stimulation (3 Hz, 1.5 mA). **b**, Representative examples of ASL perfusion weighted (control-label) images illustrating specific increases in CBF in the S1FL region of the somatosensory cortex, contralateral to the stimulated forepaw. **c**, Changes in CBF recorded in the contralateral and ipsilateral S1FL regions in response to electrical forepaw stimulation at baseline conditions and after systemic acetazolamide treatment. Specific CBF increases induced by activation of somatosensory pathways are only observed in the contralateral cortex. Data are presented as mean values ±SEM. **d**, Summary data illustrating changes in CBF recorded in the contralateral and ipsilateral S1FL regions in response to electrical forepaw stimulation at baseline conditions and after systemic acetazolamide treatment. In the box-and-whisker plots the central dot indicates the mean, the central line indicates the median, the box limits indicate the upper and lower quartiles, and the whiskers show the minimum-maximum range of the data. *P* values, two-sided Wilcoxon's matched-pairs signed rank test. Source data are provided as a Source Data file.

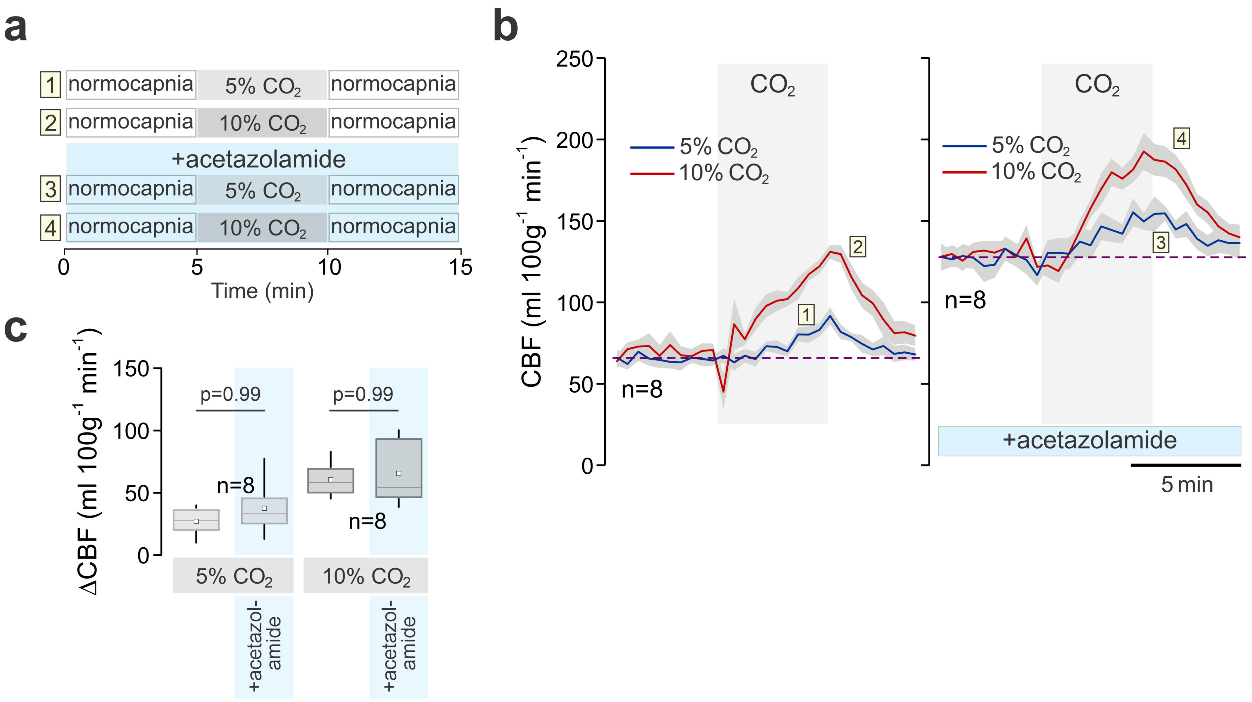

Supplementary Figure 2| Cerebrovascular reactivity to CO_2 is not affected by systemic carbonic anhydrase inhibition with acetazolamide in rats. **a**, Schematic depiction of the experimental timeline. CO_2 challenge (5% or 10% CO_2 in the inspired air) was given for 5 min before and after systemic administration of acetazolamide (10 mg kg^{-1}). **b**, Time-course of the whole brain CBF changes recorded using arterial spin labelling MRI in anaesthetised rats at resting conditions and in response to 5% and 10% inspired CO_2, applied before and after systemic carbonic anhydrase inhibition with acetazolamide. Numbers refer to the experimental conditions depicted on the schematic shown in **a**. Data are presented as mean values ±SEM. **c**, Summary data illustrating peak absolute CO_2-induced increases in CBF from the baseline before and after administration of acetazolamide. In the box-and-whisker plot the central dot indicates the mean, the central line indicates the median, the box limits indicate the upper and lower quartiles and the whiskers show the minimum-maximum range of the data. *P* values, two-sided Mann Whitney-U test. Source data are provided as a Source Data file.

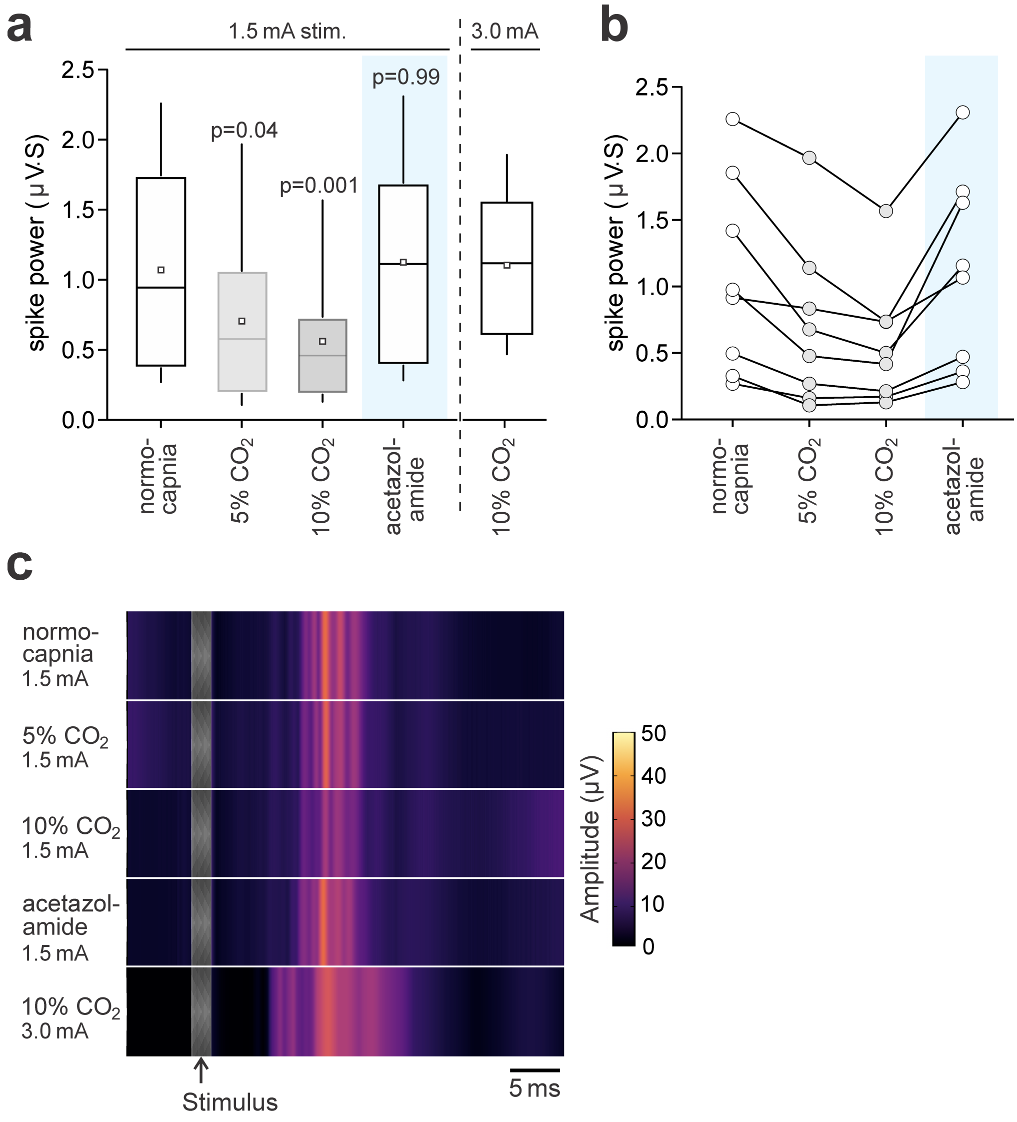

Supplementary Figure 3| CO_2 reduces neuronal excitability in the somatosensory cortex. **a**, **b**, Summary data (box and whisker plots and individual data, n=8) illustrating the effect of 5% and 10% inspired CO_2 and systemic carbonic anhydrase inhibition with acetazolamide (10 mg kg^{-1}) on the evoked neuronal responses (expressed as spike power) in the S1FL region of the somatosensory cortex induced by electrical forepaw stimulation in anaesthetised rats. In the box-and-whisker plot the central dot indicates the mean, the central line indicates the median, the box limits indicate the upper and lower quartiles and the whiskers show the minimum-maximum range of the data. *P* values, Kruskal-Wallis test followed by Dunn's multiple comparison test. **c**, False colour plots of averaged evoked extracellular potential amplitude changes recorded in the somatosensory cortex in the experimental conditions indicated. Evoked extracellular potentials were rectified and the stimulus-triggered averages were calculated over three separate trains of electrical forepaw stimulation. Acetazolamide decreased brain tissue pH but had no effect on the neuronal activity, suggesting that the inhibitory effect of CO_2 is independent of its effect on brain pH. Source data are provided as a Source Data file.

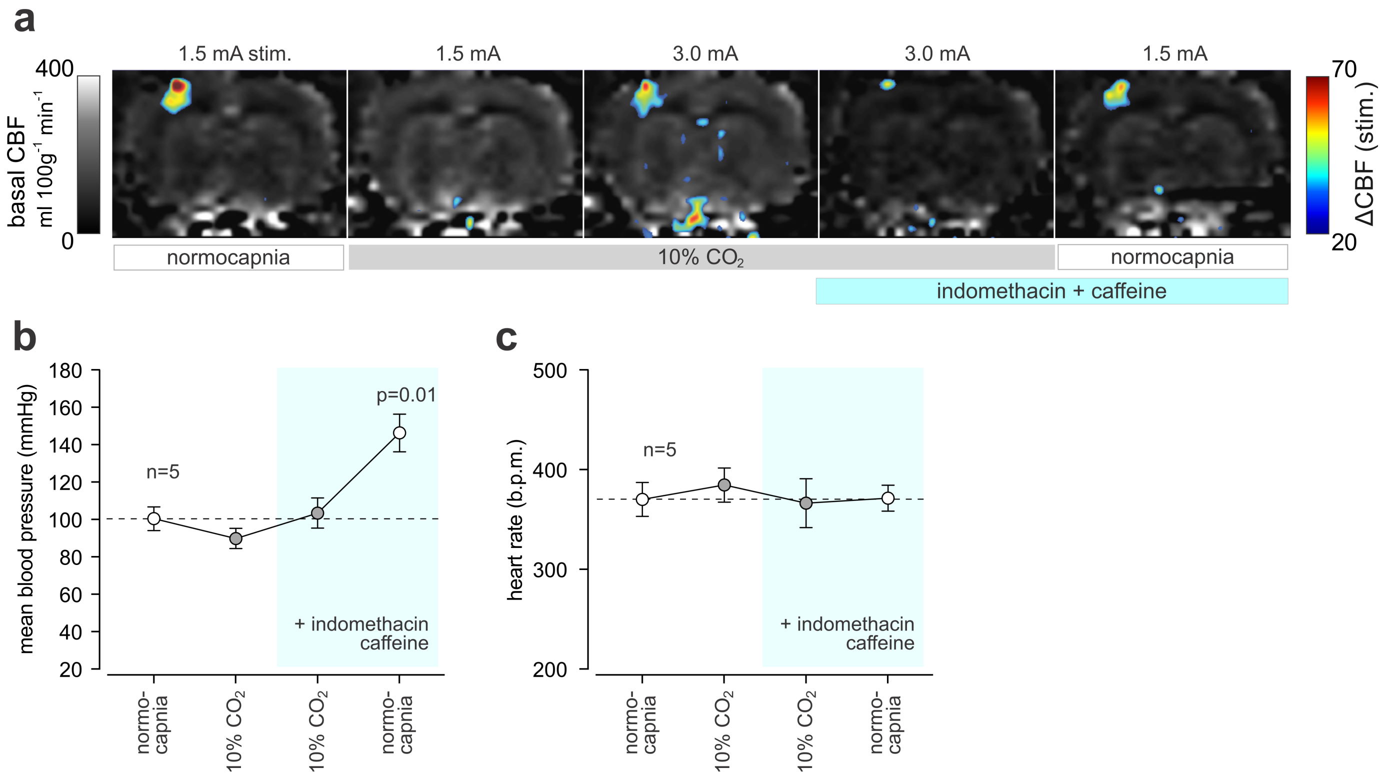

Supplementary Figure 4| **a**, Representative arterial spin labelling images of the rat brain illustrating CBF at baseline (normocapnia), in conditions of 10% inspired CO_2, after the systemic administration of indomethacin and caffeine (both at 10 mg kg^{-1}; i.v.) in conditions of 10% inspired CO_2, and after the withdrawal of inspired CO_2. Overlaid (false colour scale) illustrates CBF responses in the S1FL region of the somatosensory cortex induced by electrical forepaw stimulation (3 Hz, 1.5 or 3 mA). **b**, Summary data illustrating changes in mean arterial blood pressure and heart rate recorded in 5 animals in response to 10% inspired CO_2, after the systemic administration of indomethacin and caffeine (both at 10 mg kg^{-1}; i.v.) in conditions of 10% inspired CO_2, and after the withdrawal of inspired CO_2. Data are presented as mean values ±SEM. *P* value, one-way ANOVA followed by Holm-Sidak multiple comparison test. Source data are provided as a Source Data file.

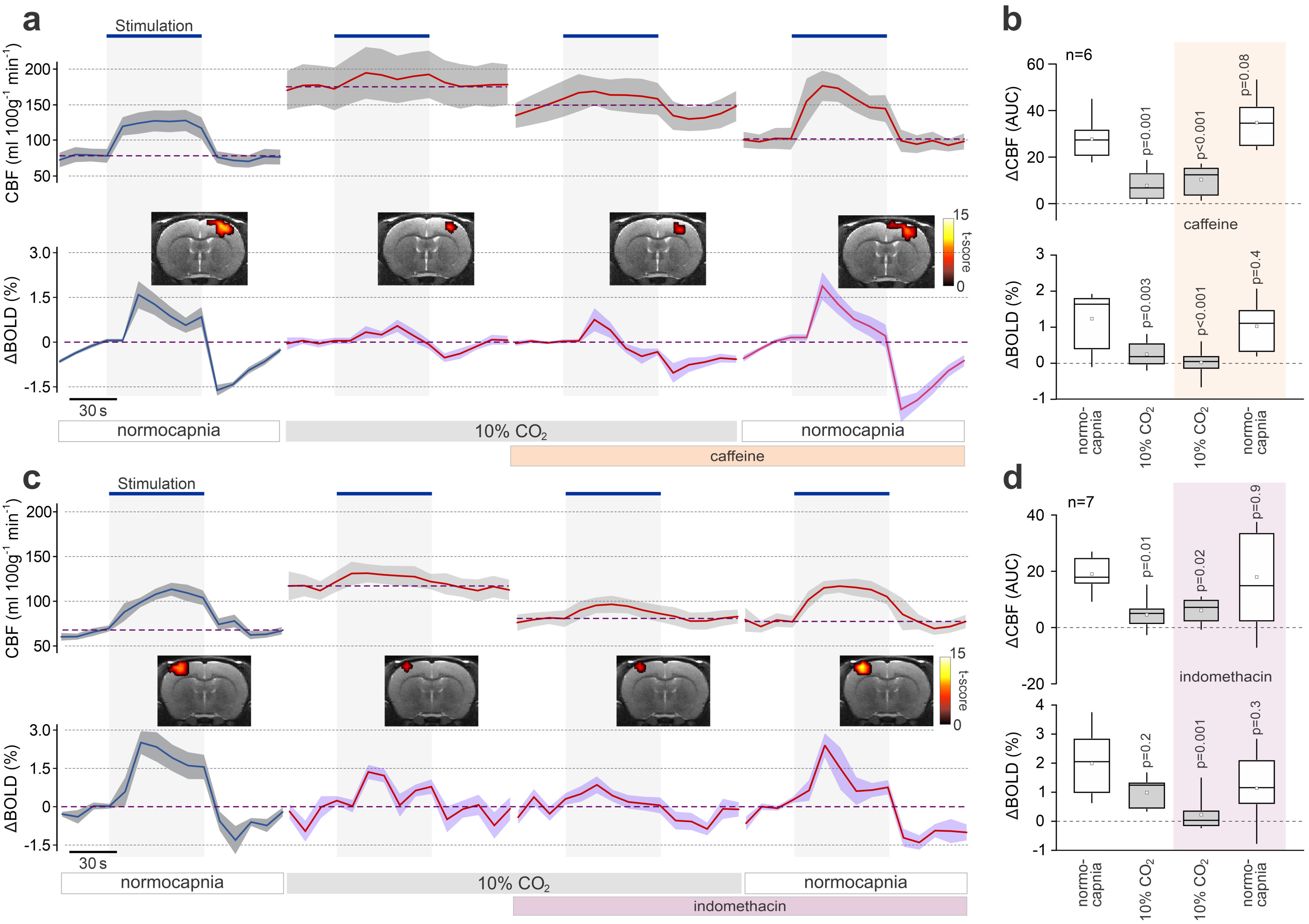

Supplementary Figure 5| CO_2 added to the inspired air prevents the development of the neurovascular response in the somatosensory cortex independently of basal CBF in rats. **a**, CBF and blood oxygen level dependent (BOLD) responses in the S1FL region induced by electrical forepaw stimulation at baseline, in conditions of 10% inspired CO_2, after the systemic administration of caffeine (10 mg kg^{-1}; i.v.) in conditions of 10% inspired CO_2, and after the withdrawal of inspired CO_2. **b,** Summary data illustrating integral CBF and peak BOLD responses in the S1FL region induced by electrical forepaw stimulation at baseline, in conditions of 10% inspired CO_2, after the systemic administration of caffeine in conditions of 10% inspired CO_2, and after the withdrawal of inspired CO_2. **c**, CBF and BOLD responses in the S1FL region induced by electrical forepaw stimulation at baseline, in conditions of 10% inspired CO_2, after the systemic administration of indomethacin (10 mg kg^{-1}; i.v.) in conditions of 10% inspired CO_2, and after the withdrawal of inspired CO_2. **d,** Summary data illustrating integral CBF and peak BOLD responses in the S1FL region induced by electrical forepaw stimulation at baseline, in conditions of 10% inspired CO_2, after the systemic administration of indomethacin in conditions of 10% inspired CO_2, and after the withdrawal of inspired CO_2. Activation maps illustrate mean BOLD signal changes in response to forepaw stimulation. Colour bars: *t*-score from SPM mixed-effects analysis, $p<0.05$ (uncorrected). In **a** and **c**, the data are presented as mean values ± SEM (shaded areas denote error bands). In the box-and-whisker plots (**b** and **d**) the central dot indicates the mean, the central line indicates the median, the box limits indicate the upper and lower quartiles, and the whiskers show the minimum-maximum range of the data. *P* values, Kruskal-Wallis test followed by Dunn's multiple comparison test. Source data are provided as a Source Data file.

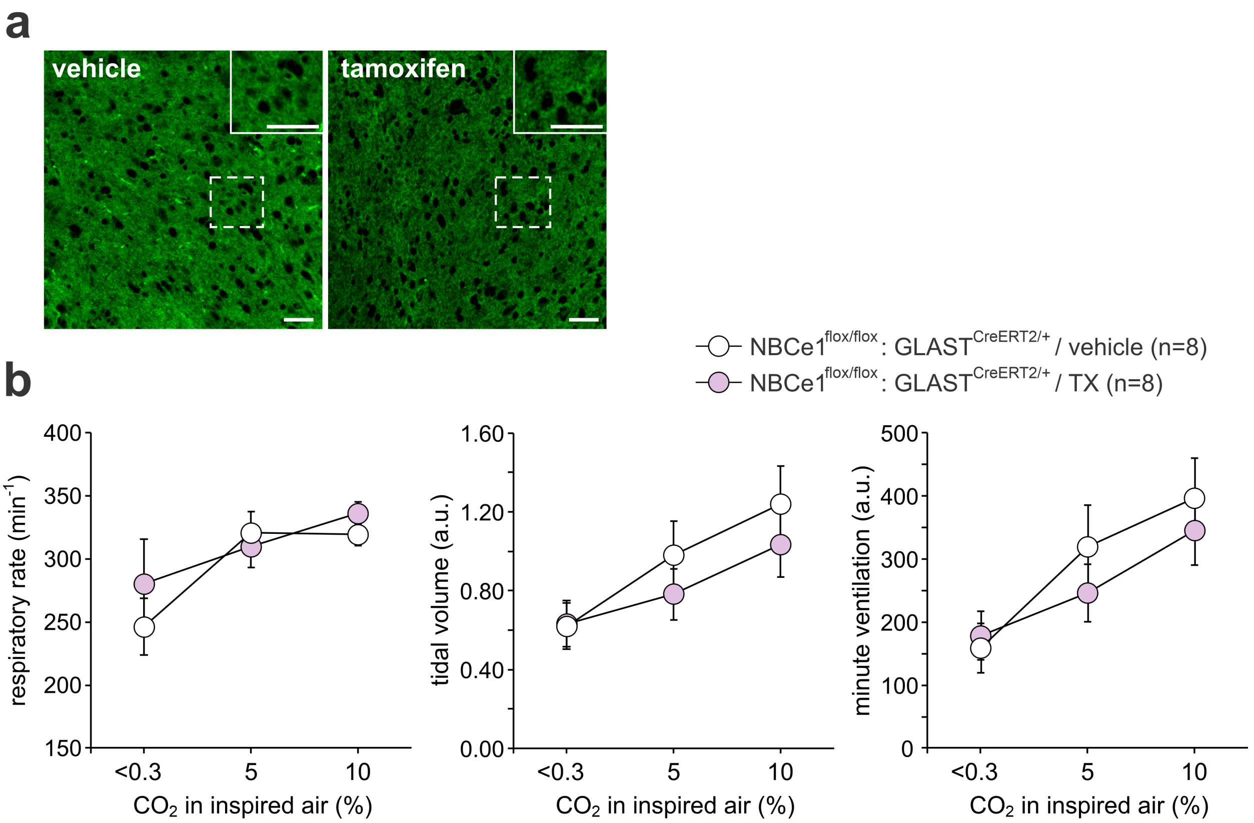

Supplementary Figure 6| NBCe1 expression in the brainstem, resting ventilation and respiratory responses to CO_2 are not affected in NBCe1$^{flox/flox}$:GLAST$^{CreERT2/+}$ mice treated with tamoxifen. **a**, Representative confocal images illustrating immunohistochemical detection of NBCe1 in the brainstem of NBCe1$^{flox/flox}$:GLAST$^{CreERT2/+}$ mice treated with the vehicle (oil) or tamoxifen (repeated in 4 animals from each experimental group with similar result). In contrast to reduced expression of NBCe1 in the cortex, tamoxifen treatment of NBCe1$^{flox/flox}$:GLAST$^{CreERT2/+}$ mice had no effect on NBCe1 expression in the brainstem. Scale bars = 50 μm. **b,** Ventilatory responses to 5% and 10% inspired CO_2 in NBCe1$^{flox/flox}$:GLAST$^{CreERT2/+}$ mice treated with the vehicle or tamoxifen (TX). Data are presented as mean values ±SEM. Numbers in parentheses indicate sample sizes. Source data are provided as a Source Data file.

Supplementary Table 1

Study	Species	Method	Pathway(s) targeted	Drug(s) used or gene deleted
Heinert et al. 1999[37]	Rat	Hydrogen clearance (CBF)	COX + NOS	Indomethacin + 7-NI
Wang et al. 1994[115]	Rat	Radioactivity clearance (CBF)	COX + NOS	Indomethacin + L-NA
Xu et al. 2004[126]	Rat	Cranial window (vessel diameter)	COX + NOS	Indomethacin + L-NNA
Xu et al. 2004[126]	Rat	Cranial window (vessel diameter)	COX + NOS	Indomethacin + L-NNA + L-αAAA
Zhang et al. 2002[130]	Pig	Cranial window (vessel diameter)	COX + NOS	Indomethacin + L-NAME
Zuckerman et al. 1996[131]	Pig	Cranial window (vessel diameter)	COX + NOS	Indomethacin + L-NA
Howarth et al. 2017[45]	Rat	Laser doppler (CBF)	COX-1	SC-560
Niwa et al. 2001[75]	Mouse	Radioactivity clearance (CBF)	COX-1	SC-560
Niwa et al. 2001[75]	Mouse	Radioactivity clearance (CBF)	COX-1	COX-1 knockout
Uekawa et al. 2016[106]	Mouse	Laser doppler (CBF)	COX-1	SC-560
Bakalova et al. 2002[1]	Rat	Laser doppler (CBF)	COX	Indomethacin
Barnes et al. 2012[2]	Human	Doppler Ultrasound (CBF)	COX	Indomethacin
Busija et al. 1983[9]	Cat	Microspheres (CBF)	COX	Indomethacin
Busija et al. 1983[10]	Rabbit	Microspheres (CBF)	COX	Indomethacin
Csete et al. 2001[13]	Rabbit	Hydrogen clearance (CBF)	COX	Indomethacin
Cuypers et al. 1978[14]	Rabbit	Thermomonitoring (CBF)	COX	Indomethacin
Dahlgren et al. 1981[15]	Rat	Kety-Schmidt technique (CBF)	COX	Indomethacin
Dahlgren et al. 1981[18]	Rat	Kety-Schmidt technique (CBF)	COX	Indomethacin
Eriksson et al. 1983[23]	Human	Kety-Schmidt technique (CBF)	COX	Indomethacin
Fan et al. 2010[27]	Human	Doppler Ultrasound (CBF)	COX	Indomethacin
Gabrielyan et al. 1979[30]	Cat	Hydrogen clearance (CBF)	COX	Indomethacin
Gabrielyan et al. 1979[30]	Dog	Electromagnetic flowmeter (CBF)	COX	Indomethacin
Heinert et al. 1998[36]	Rat	Hydrogen clearance (CBF)	COX	Indomethacin
Heinert et al. 1999[37]	Rat	Hydrogen clearance (CBF)	COX	Indomethacin
Hoffman et al. 1982[40]	Goat	Electromagnetic flowmeter (CBF)	COX	Indomethacin
Hoiland et al. 2015[42]	Human	Doppler Ultrasound (CBF)	COX	Indomethacin

Hoiland et al. 2016[41]	Human	Doppler Ultrasound (CBF)	COX	Indomethacin
Iadecola et al. 1994[51]	Rat	Laser doppler (CBF)	COX	Indomethacin
Ivancev et al. 2009[56]	Human	Doppler Ultrasound (CBF)	COX	Indomethacin
Jackson et al. 1983[57]	Dog	Microspheres (CBF)	COX	Indomethacin
Kastrup et al. 1999[59]	Human	Doppler Ultrasound (CBF)	COX	Indomethacin
Leffler et al. 1993[63]	Pig	Cranial window (vessel diameter)	COX	Indomethacin
Leffler et al. 1994[64]	Pig	Cranial window (vessel diameter)	COX	Indomethacin
Markus et al. 1994[66]	Human	Doppler Ultrasound (CBF)	COX	Indomethacin
McCalden et al. 1984[67]	Baboon	Radioactivity clearance (CBF)	COX	Indomethacin
Parfenova et al. 1994[79]	Pig	Cranial window (vessel diameter)	COX	Indomethacin
Peltonen et al. 2015[83]	Human	Doppler Ultrasound (CBF)	COX	Indomethacin
Peltonen et al. 2016[82]	Human	Doppler Ultrasound (CBF)	COX	Indomethacin
Pickard et al. 1973[87]	Baboon	Radioactivity clearance (CBF)	COX	Indomethacin
Pickard et al. 1980[88]	Baboon	Radioactivity clearance (CBF)	COX	Indomethacin
Pickles et al. 1984[89]	Human	Radioactivity clearance (CBF)	COX	Indomethacin
Pourcyrous et al. 1999[90]	Pig	Microspheres (CBF)	COX	Indomethacin
Quintana et al. 1988[91]	Rat	Microspheres (CBF)	COX	Indomethacin
Sakabe et al. 1979[95]	Rat	Kety-Schmidt technique (CBF)	COX	Indomethacin
Shoemaker et al. 2021[99]	Human	Doppler Ultrasound (CBF)	COX	Indomethacin
St Lawrence et al. 2002[103]	Human	MRI (CBF)	COX	Indomethacin
Vuletic et al. 2013[107]	Human	Doppler Ultrasound (CBF)	COX	Indomethacin
Wagerle et al. 1988[108]	Pig	Cranial window (vessel diameter)	COX	Indomethacin
Wang et al. 1993[112]	Rat	Radioactivity clearance (CBF)	COX	Indomethacin
Wang et al. 1994[115]	Rat	Radioactivity clearance (CBF)	COX	Indomethacin
Wang et al. 1999[110]	Rat	Cranial window (vessel diameter)	COX	Indomethacin
Wei et al. 1980[116]	Cat	Cranial window (vessel diameter)	COX	Indomethacin
Wennmalm et al. 1981[118]	Human	Kety-Schmidt technique (CBF)	COX	Indomethacin
Wennmalm et al. 1984[117]	Human	Kety-Schmidt technique (CBF)	COX	Indomethacin
Xie et al. 2006[125]	Human	Doppler Ultrasound (CBF)	COX	Indomethacin

Xie et al. 2009[124]	Human	Doppler Ultrasound (CBF)	COX	Indomethacin
Zhang et al. 2002[130]	Pig	Cranial window (vessel diameter)	COX	Indomethacin
Zuckerman et al. 1996[131]	Pig	Cranial window (vessel diameter)	COX	Indomethacin
Bonnin et al. 2020[6]	Mouse	Doppler Ultrasound (CBF)	nNOS	7-NI
Fabricius et al. 1996[26]	Rat	Laser doppler (CBF)	nNOS	7-NI
Harada et al. 1997[33]	Rat	Laser doppler (CBF)	nNOS	7-NI
Heinert et al. 1998[36]	Rat	Hydrogen clearance (CBF)	nNOS	7-NI
Heinert et al. 1999[37]	Rat	Hydrogen clearance (CBF)	nNOS	7-NI
Iadecola et al. 1996[49]	Rat	Laser doppler (CBF)	nNOS	7-NI
Irikura et al. 1995[53]	Mouse	Laser doppler (CBF)	nNOS	nNOS knockout
Najarian et al. 2000[73]	Pig	Microspheres (CBF)	nNOS	TRIM
Najarian et al. 2000[73]	Pig	Microspheres (CBF)	nNOS	Br-7-NI
Okamoto et al. 1997[78]	Rat	Laser doppler (CBF)	nNOS	7-NI
Smith et al. 1997[102]	Rat	Laser doppler (CBF)	nNOS	7-NI
Wang et al. 1998[109]	Rat	Cranial window (vessel diameter)	nNOS	7-NI
Wang et al. 1999[110]	Rat	Cranial window (vessel diameter)	nNOS	7-NI
Wang et al. 1995[113]	Rat	Laser doppler (CBF)	nNOS	7-NI
Xu et al. 2004[126]	Rat	Cranial window (vessel diameter)	nNOS	ARR-17477
Yang et al. 2003[127]	Mouse	Laser doppler (CBF)	nNOS	nNOS knockout
Zhang et al. 1995[129]	Rat	Laser doppler (CBF)	nNOS	7-NI
Zhang et al. 2002[130]	Pig	Cranial window (vessel diameter)	nNOS	7-NI
Bonnin et al. 2020[6]	Mouse	Doppler Ultrasound (CBF)	NOS	L-NIO
Bonnin et al. 2020[6]	Mouse	Doppler Ultrasound (CBF)	NOS	L-NMMA
Bonvento et al. 1994[7]	Rat	Radioactivity clearance (CBF)	NOS	L-NAME
Buchanan et al. 1993[8]	Rats	Retroglenoid outflow (CBF)	NOS	L-NAME
Chandra et al. 2016[11]	Mouse	ASL MRI (CBF)	NOS	eNOS knockout
Csete et al. 2001[13]	Rabbit	Hydrogen clearance (CBF)	NOS	L-NAME
Dieguez et al. 1993[19]	Goat	Electromagnetic flowmeter (CBF)	NOS	L-NAME
Estevez et al. 1997[24]	Rat	Laser doppler (CBF)	NOS	L-NAME

Fabricius et al. 1994[25]	Rat	Laser doppler (CBF)	NOS	L-NNA
Fabricius et al. 1996[26]	Rat	Laser doppler (CBF)	NOS	L-NNA
Faraci et al. 1994[29]	Rabbit	Cranial window (vessel diameter)	NOS	L-NNA
Faraci et al. 2019[28]	Mouse	Cranial window (vessel diameter)	NOS	L-NNA
Goadsby et al. 1994[31]	Cat	Laser doppler (CBF)	NOS	L-NAME
Harris et al. 2008[35]	Sheep	Laser doppler (CBF)	NOS	L-NAME
Heinert et al. 1998[36]	Rat	Hydrogen clearance (CBF)	NOS	L-NMMA
Horvath et al. 1994[43]	Rat	Kety-Schmidt technique (CBF)	NOS	L-NAME
Hudetz et al.1995[46]	Rat	Laser doppler (CBF)	NOS	L-NAME
Iadecola et al.1993[50]	Rat	Laser doppler (CBF)	NOS	L-NAME
Iadecola et al.1994[48]	Rat	Laser doppler (CBF)	NOS	L-NAME
Iadecola et al. 1994[51]	Rat	Laser doppler (CBF)	NOS	L-NAME
Iadecola et al. 1994[47]	Rat	Laser doppler (CBF)	NOS	L-NA
Iadecola et al. 1996[49]	Rat	Laser doppler (CBF)	NOS	L-NNA
Ide et al. 2007[52]	Human	Doppler Ultrasound (CBF)	NOS	L-NMMA
Irikura et al. 1994[54]	Rat	Laser doppler (CBF)	NOS	L-NNA
Irikura et al. 1995[53]	Mouse	Laser doppler (CBF)	NOS	nNOS knockout + L-NNA
Komjati et al. 2001[60]	Cat	Microspheres (CBF)	NOS	L-NAME
Komjati et al. 2001[60]	Cat	Microspheres (CBF)	NOS	L-NAME
Ma et al. 1996[65]	Mouse	Laser doppler (CBF)	NOS	L-NNA
McPhearson et al. 1995[68]	Macaque	Microspheres (CBF)	NOS	L-NAME
Meng et al. 1995[69]	Pig	Cranial window (vessel diameter)	NOS	L-NNA
Najarian et al. 2000[73]	Pig	Microspheres (CBF)	NOS	L-NA
Niwa et al. 1993[76]	Rat	Laser doppler (CBF)	NOS	L-NA
Parfenova et al. 1994[79]	Pig	Cranial window (vessel diameter)	NOS	L-NAME
Parfenova et al. 1994[79]	Pig	Cranial window (vessel diameter)	NOS	L-NMMA
Parfenova et al. 1994[79]	Pig	Cranial window (vessel diameter)	NOS	L-NNA
Pelligrino et al. 1993[80]	Rat	Radioactivity clearance (CBF)	NOS	L-NAME
Phillis et al. 2003[85]	Rat	Laser doppler (CBF)	NOS	L-NAME

Phillis et al. 2004[86]	Rat	Cranial window (vessel diameter)	NOS	L-NAME
Phillis et al. 2004[86]	Rat	Cranial window (vessel diameter)	NOS	L-NA
Reid et al. 1995[92]	Rat	Hydrogen clearance (CBF)	NOS	L-NMMA
Sandor et al. 1994[96]	Cat	Radioactivity clearance (CBF)	NOS	L-NAME
Schmetterer et al. 1997[97]	Human	Doppler Ultrasound (CBF)	NOS	L-NMMA
Smith et al. 1997[102]	Rat	Laser doppler (CBF)	NOS	L-NAME
Thompson et al. 1996[105]	Macaque	Thermomonitoring (CBF)	NOS	L-NMMA
Thompson et al. 1996[105]	Macaque	Thermomonitoring (CBF)	NOS	NLA
Wang et al. 1992[111]	Rat	Radioactivity clearance (CBF)	NOS	L-NNA
Wang et al. 1994[114]	Rat	Cranial window (vessel diameter)	NOS	L-NA
Wang et al. 1994[115]	Rat	Radioactivity clearance (CBF)	NOS	L-NA
White et al.1998[119]	Human	Doppler Ultrasound (CBF)	NOS	L-NMMA
Wolk et al. 1995[122]	Rat	Laser doppler (CBF)	NOS	L-NAME
Wolk et al. 1995[123]	Rat	Laser doppler (CBF)	NOS	L-NAME
Wolk et al. 1995[123]	Rat	Laser doppler (CBF)	NOS	L-NAME
Xu et al. 2004[126]	Rat	Cranial window (vessel diameter)	NOS	L-NNA
Xu et al. 2004[126]	Rat	Cranial window (vessel diameter)	NOS	L-NNA + L-αAAA
Zhang et al. 1995[129]	Rat	Laser doppler (CBF)	NOS	L-NA
Zhang et al. 2002[130]	Pig	Cranial window (vessel diameter)	NOS	L-NAME
Zuckerman et al. 1996[131]	Pig	Cranial window (vessel diameter)	NOS	L-NA
Kannurpratti et al. 2008[58]	Rat	Laser doppler (CBF)	Ca^{2+} channels	Ruthenium red
Kannurpratti et al. 2008[58]	Rat	Laser doppler (CBF)	Ca^{2+} channels	Ru360
Nnorom et al. 2014[77]	Pig	Cranial window (vessel diameter)	Ca^{2+} channels	Paxilline
Simpson III et al. 1991[101]	Rat	Retroglenoid outflow (CBF)	Ca^{2+} channels	Nifedipine
Takenaka et al. 2003[104]	Rabbit	Cranial window (vessel diameter)	Ca^{2+} channels	Nicardipine
Wang et al. 1998[109]	Rat	Cranial window (vessel diameter)	Ca^{2+} channels	Iberiotoxin
Heinonen et al. 2003[38]	Pig	Cranial window (vessel diameter)	Prostaglandin signaling	Dexamethasone
Hoffman et al. 1982[40]	Goat	Electromagnetic flowmeter (CBF)	Prostaglandin signaling	Ibuprofen
Uekawa et al. 2016[106]	Mouse	Laser doppler (CBF)	Prostaglandin signaling	SC-51089

Uekawa et al. 2016[106]	Mouse	Laser doppler (CBF)	Prostaglandin signaling	EP1 knockout
Uekawa et al. 2016[106]	Mouse	Laser doppler (CBF)	Prostaglandin signaling	L-798106
Uekawa et al. 2016[106]	Mouse	Laser doppler (CBF)	Prostaglandin signaling	ONO-AE3-208
Wei et al. 1980[116]	Cat	Cranial window (vessel diameter)	Prostaglandin signaling	AHR-5850
Blaha et al. 2007[5]	Human	Doppler Ultrasound (CBF)	Adenosine signaling	caffeine
Chen et al. 2009[12]	Human	MRI (CBF)	Adenosine signaling	caffeine
Estevez et al. 1997[24]	Rat	Laser doppler (CBF)	Adenosine signaling	caffeine
Estevez et al. 1997[24]	Rat	Laser doppler (CBF)	Adenosine signaling	CGS 15943
Hoffman et al. 1984[39]	Rat	Microspheres (CBF)	Adenosine signaling	Theophylline
Ito et al. 1999[55]	Human	PET (CBF)	Adenosine signaling	Dipyridamole
Meno et al. 2001[70]	Rat	Cranial window (vessel diameter)	Adenosine signaling	8-SPT
Meno et al. 2001[70]	Rat	Cranial window (vessel diameter)	Adenosine signaling	Theophylline
Morii et al. 1987[71]	Rat	Retroglenoid outflow (CBF)	Adenosine signaling	Theophylline
Phillis et al. 1987[84]	Rat	Retroglenoid outflow (CBF)	Adenosine signaling	Caffeine
Phillis et al. 2003[85]	Rat	Laser doppler (CBF)	Adenosine signaling	Caffeine
Phillis et al. 2003[85]	Rat	Laser doppler (CBF)	Adenosine signaling	ZM 241385
Phillis et al. 2004[86]	Rat	Cranial window (vessel diameter)	Adenosine signaling	ZM 241385
Phillis et al. 2004[86]	Rat	Cranial window (vessel diameter)	Adenosine signaling	SCH58261
Simpson et al. 1991[100]	Rat	Cranial window (vessel diameter)	Adenosine signaling	Adenosine deaminase
Williams et al. 1991[120]	Rat	Radioactivity clearance (CBF)	Adenosine signaling	Phentolamine
Parfenova et al. 1994[79]	Pig	Cranial window (vessel diameter)	cGMP	LY 83583
Parfenova et al. 1994[79]	Pig	Cranial window (vessel diameter)	cGMP	Methylene blue
Rosenblum et al. 2002[94]	Rat	Cranial window (vessel diameter)	cGMP	ODQ
Willis et al. 2001[121]	Pig	Cranial window (vessel diameter)	cGMP	ODQ
Kontos et al. 1984[61]	Cat	Cranial window (vessel diameter)	Multiple (free radicals)	Superoxide dismutase + catalase
Nnorom et al. 2014[77]	Pig	Cranial window (vessel diameter)	Multiple (K_{ATP} + calcium channels)	Glibenclamide + Paxilline
Pelligrino et al. 1995[81]	Rat	Cranial window (vessel diameter)	Multiple (adenosine signaling + NMDA receptors)	8-SPT + MK-801

Reid et al. 1995[92]	Rat	Hydrogen clearance (CBF)	Multiple (NOS + K^+ channels)	Glibenclamide + L-NMMA
Scremin et al. 1982[98]	Rabbit	Electromagnetic flowmeter (CBF)	Multiple (muscarinic)	Physostigime + dihydrobetaerythroidine
Scremin et al. 1982[98]	Rabbit	Electromagnetic flowmeter (CBF)	Multiple (muscarinic)	Physostigime + atropine
Wagerle et al. 1988[108]	Pig	Cranial window (vessel diameter)	Multiple (COX + adenosine signaling)	Indomethacin + Theophylline
Zhang et al. 1998[128]	Rat	Laser doppler (CBF)	Multiple (homocyteine + ROS)	Homocyteine + superoxide dismutase
Domoki et al. 1999[20]	Pig	Cranial window (vessel diameter)	COX	Cycloheximide
Eriksson et al. 1983[23]	Human	Kety-Schmidt technique (CBF)	COX	Naproxen
Eriksson et al. 1983[23]	Human	Kety-Schmidt technique (CBF)	COX	Aspirin
Hoiland et al. 2016[41]	Human	Doppler Ultrasound (CBF)	COX	Naproxen
Hoiland et al. 2016[41]	Human	Doppler Ultrasound (CBF)	COX	Ketorolac
Markus et al. 1994[66]	Human	Doppler Ultrasound (CBF)	COX	Aspirin
Markus et al. 1994[66]	Human	Doppler Ultrasound (CBF)	COX	Sulindac
Najarian et al. 2000[73]	Pig	Microspheres (CBF)	COX	Diclofenac
Quintana et al. 1988[91]	Rat	Microspheres (CBF)	COX	Diclofenac
Wang et al. 1993[112]	Rat	Radioactivity clearance (CBF)	COX	Diclofenac
Wennmalm et al. 1984[117]	Human	Kety-Schmidt technique (CBF)	COX	Naproxen
Wennmalm et al. 1984[117]	Human	Kety-Schmidt technique (CBF)	COX	Aspirin
Berntman et al. 1979[4]	Rat	Kety-Schmidt technique (CBF)	Catecholamines	Propranolol
Berntman et al. 1979[4]	Rat	Kety-Schmidt technique (CBF)	Catecholamines	Diazepam
Dahlgren et al. 1981[16]	Rat	Kety-Schmidt technique (CBF)	Catecholamines	5,7-DHT
Dahlgren et al. 1981[17]	Rat	Kety-Schmidt technique (CBF)	Catecholamines	Lesion of Locus coeruleus
Dahlgren et al. 1981[16]	Rat	Radioactivity clearance (CBF)	Catecholamines	6-OHDA
Edvinsson et al. 1977[22]	Rat	Radioactivity clearance (CBF)	Catecholamines	6-OHDA
Harik et al. 1986[34]	Rat	Radioactivity clearance (CBF)	Catecholamines	Lesion of *locus coeruleus*
Wagerle et al. 1988[108]	Pig	Cranial window (vessel diameter)	Arachidonic acid synthesis	Nordihydroguaiaretic
Wagerle et al. 1988[108]	Pig	Cranial window (vessel diameter)	Arachidonic acid synthesis	Quinacrine
Wagerle et al. 1988[108]	Pig	Cranial window (vessel diameter)	Arachidonic acid synthesis	p-Bromophenacyl Bromide
Nagayama et al. 1998[72]	Mouse	Laser doppler (CBF)	COX-2	NS-398

Niwa et al. 2000[74]	Mouse	Laser doppler (CBF)	COX-2	COX-2 knockout
Niwa et al. 2000[74]	Mouse	Laser doppler (CBF)	COX-2	NS-398
Niwa et al. 2000[74]	Mouse	Laser doppler (CBF)	COX-2	COX-2 knockout + NS-398
Uekawa et al. 2016[106]	Mouse	Laser doppler (CBF)	COX-2	NS-398
Bayerle-Eder et al. 2000[3]	Human	Doppler Ultrasound (CBF)	K^+ channels	Glibenclamide
Domoki et al. 2005[21]	Pig	Cranial window (vessel diameter)	K^+ channels	5-hydroxydecanoate
Faraci et al. 1994[29]	Rabbit	Cranial window (vessel diameter)	K^+ channels	Glibenclamide
Hosford et al. 2019[44]	Mouse	ASL MRI (CBF)	K^+ channels	Kir6.1 knockout
Nnorom et al. 2014[77]	Pig	Cranial window (vessel diameter)	K^+ channels	Glibenclamide
Reid et al. 1993[93]	Rat	Hydrogen clearance (CBF)	K^+ channels	Tolbutamide
Wang et al. 1998[109]	Rat	Cranial window (vessel diameter)	K^+ channels	Glibenclamide
Hansen et al. 2018[32]	Human	Doppler Ultrasound (CBF)	ROS	Antioxidant cocktail (Vitamin C, Vitamin E, α-lipoic acid)
Leffler et al. 1991[62]	Pig	Cranial window (vessel diameter)	ROS	Superoxide dismutase + catalase
Leffler et al. 1991[62]	Pig	Cranial window (vessel diameter)	ROS	Tiron
Leffler et al. 1991[62]	Pig	Cranial window (vessel diameter)	ROS	N-2-mercaptopropionyl glycine (MPG)
Niwa et al. 2001[75]	Mouse	Laser doppler (CBF)	ROS	Superoxide dismutase
Wagerle et al. 1988[108]	Pig	Cranial window (vessel diameter)	ROS	Superoxide dismutase and catalase

Abbreviations: 5,7-DHT, 5,7-dihydroxytryptamine; 6-OHDA, 6-hydroxydopamine; 7-NI, 7-nitroindazole; 8-SPT, 8-(p-Sulfophenyl)theophylline; BR-7-NI, 3-bromo-7- nitroindazole; CBF, cerebral blood flow; COX, cyclooxygenase; eNOS, endothelial nitric oxide synthase; L-NA/NLA, N^G-Nitro-L-arginine; L-NAME, N^ω-nitro-L-arginine methyl ester; L-NMMA, *N*-monomethyl-L-arginine; L-NNA, N^ω-nitro-L- arginine; L-αAAA, L-α-aminoadipic acid; nNOS, neuronal nitric oxide synthase; NOS, nitric oxide synthase; ODQ, 1H-[1,2,4]oxadiazolo[4,3,-a]quinoxalin-1-one; TRIM, 1-(2- trifluoromethylphenyl) imidazole.

Supplementary References

1. Bakalova, R. A., Matsuura, T. & Kanno, I. Cyclooxygenase-pathway participates in the regulation of regional cerebral blood flow in response to neuronal activation under normo- and hypercapnia. *Prostaglandins Leukot. Essent. Fat. Acids* **67**, 379–388 (2002).
2. Barnes, J. N., Schmidt, J. E., Nicholson, W. T. & Joyner, M. J. Cyclooxygenase inhibition abolishes age-related differences in cerebral vasodilator responses to hypercapnia. *J. Appl. Physiol.* **112**, 1884–1890 (2012).
3. Bayerle-Eder, M. et al. Hypercapnia-induced cerebral and ocular vasodilation is not altered by glibenclamide in humans. *Am. J. Physiol. - Regul. Integr. Comp. Physiol.* **278**, 1667–1673 (2000).
4. Berntman, L., Dahlgren, N. & Siesjö, B. K. Cerebral blood flow and oxygen consumption in the rat brain during extreme hypercarbia. *Anesthesiology* **50**, 299–305 (1979).
5. Blaha, M., Benes, V., Douville, C. M. & Newell, D. W. The effect of caffeine on dilated cerebral circulation and on diagnostic CO_2 reactivity testing. *J. Clin. Neurosci.* **14**, 464–467 (2007).
6. Bonnin, P. et al. Cerebral vasodilator property of poly(Adp-ribose) polymerase inhibitor (pj34) in the neonatal and adult mouse is mediated by the nitric oxide pathway. *Int. J. Mol. Sci*. **21**, 1–16 (2020).
7. Bonvento, G., Seylaz, J. & Lacombe, P. Widespread attenuation of the cerebrovascular reactivity to hypercapnia following inhibition of nitric oxide synthase in the conscious rat. *J. Cereb. Blood Flow Metab*. **14**, 699–703 (1994).
8. Buchanan, J. E. & Phillis, J. W. The role of nitric oxide in the regulation of cerebral blood flow. *Brain Res*. **610**, 248–255 (1993).
9. Busija, D. W. & Heistad, D. D. Effects of indomethacin on cerebral blood flow during hypercapnia in cats. *Am. J. Physiol. - Hear. Circ. Physiol.* **13**, (1983).
10. Busija, D. W. Role of prostaglandins in the response of the cerebral circulation to carbon dioxide in conscious rabbits. *J. Cereb. Blood Flow Metab.* **3**, 376–380 (1983).
11. Chandra, S. B. et al. Targeted overexpression of endothelial nitric oxide synthase in endothelial cells improves cerebrovascular reactivity in Ins2 Akita-type-1 diabetic mice. *J. Cereb. Blood Flow Metab.* **36**, 1135–1142 (2016).
12. Chen, Y. & Parrish, T. B. Caffeine's effects on cerebrovascular reactivity and coupling between cerebral blood flow and oxygen metabolism. *Neuroimage* **44**, 647–652 (2009).
13. Csete, K., Barzó, P., Bodosi, M. & Papp, J. G. Influence of nitrovasodilators and cyclooxygenase inhibitors on cerebral vasoreactivity in conscious rabbits. *Eur. J. Pharmacol*. **412**, 301–309 (2001).
14. Cuypers, J., Cuevas, A. & Duisberg, R. Effect of indomethacin on CO_2-induced hyperaemia (CO_2-response) in the rabbit brain. *Neurochirurgia (Stuttg).* **21**, 62–66 (1978).
15. Dahlgren, N. & Siesjö, B. K. Effects of indomethacin on cerebral blood flow and oxygen consumption in barbiturate-anesthetized Normocapnic and hypercapnic rats. *J. Cereb. Blood Flow Metab*. **1**, 109–15 (1981).
16. Dahlgren, N., Lindvall, O., Nobin, A. & Stenevi, U. Cerebral circulatory response to hypercapnia: effects of lesions of central dopaminergic and serotoninergic neuron systems. *Brain Res.* **230**, 221–33 (1981).
17. Dahlgren, N., Lindvall, O., Sakabe, T., Stenevi, U. & Siesjö, B. K. Cerebral blood flow and oxygen consumption in the rat brain after lesions of the noradrenergic locus coeruleus system. *Brain Res*. **209**, 11–23 (1981).
18. Dahlgren, N., Nilsson, B., Sakabe, T. & Siesjö, B. K. The effect of indomethacin on cerebral blood flow and oxygen consumption in the rat at normal and increased carbon dioxide tensions. *Acta Physiol. Scand.* **111**, 475–85 (1981).
19. Dieguez, G. et al. Role of NO in goat basal cerebral circulation and after vasodilatation to hypercapnia or brief ischemias. *Am. J. Physiol. - Regul. Integr. Comp. Physiol*. **265**, (1993).
20. Domoki, F. et al. Cycloheximide rapidly inhibits cortical COX activity and COX-dependent pial arteriolar dilation in piglets. *Am. J. Physiol. - Hear. Circ. Physiol*. **277**, 1113–1118 (1999).
21. Domoki, F. et al. Diazoxide preserves hypercapnia-induced arteriolar vasodilation after global cerebral ischemia in piglets. *Am. J. Physiol. - Heart Circ. Physiol*. **289**, 368–373 (2005).
22. Edvinsson, L., Hardebo, J. E. & MacKenzie, E. T. Effects of intraventricular 6-Hydroxydopamine on cerebrovascular CO_2 reactivity in anesthetized rats. *Acta Physiol. Scand.* **101**, 122–125 (1977).
23. Eriksson, S. et al. Effect of prostaglandin synthesis inhibitors on basal and CO_2-stimulated cerebral blood flow in man. *Gen. Pharmacol.* **14**, 179–180 (1983).

24. Estevez, A. Y. & Phillis, J. W. Hypercapnia-induced increases in cerebral blood flow: Roles of adenosine, nitric oxide and cortical arousal. *Brain Res*. **758**, 1–8 (1997).
25. Fabricius, M. & Lauritzen, M. Examination of the role of nitric oxide for the hypercapnic rise of cerebral blood flow in rats. *Am. J. Physiol. - Hear. Circ. Physiol*. **266**, (1994).
26. Fabricius, M., Rubin, I., Bundgaard, M. & Lauritzen, M. NOS activity in brain and endothelium: Relation to hypercapnic rise of cerebral blood flow in rats. *Am. J. Physiol. - Hear. Circ. Physiol.* **271**, (1996).
27. Fan, J. L. et al. Influence of indomethacin on ventilatory and cerebrovascular responsiveness to CO2 and breathing stability: The influence of PCO_2 gradients. *Am. J. Physiol. - Regul. Integr. Comp. Physiol*. **298**, 1648–1658 (2010).
28. Faraci, F. M. et al. Acid-sensing ion channels: novel mediators of cerebral vascular responses. *Circ. Res*. **125**, 907–920 (2019).
29. Faraci, F. M., Breese, K. R. & Heistad, D. D. Cerebral vasodilation during hypercapnia. *Stroke* **25**, 1679–1683 (1994).
30. Gabrielyan, E. S., Amroyan, E. A. & Megrabyan, V. I. Responses of the cerebral blood flow to hypo-and hypercapnia after inhibition of prostaglandin biosynthesis by indomethacin. *Bull. Exp. Biol. Med.* **87**, 230–233 (1979).
31. Goadsby, P. J. Nitric oxide is not the sole determinant of hypercapnic or metabolically driven vasodilation in the cerebral circulation. *J. Auton. Nerv. Syst*. **49**, 67–72 (1994).
32. Hansen, A. B. et al. UBC-Nepal expedition: The use of oral antioxidants does not alter cerebrovascular function at sea level or high altitude. *Exp. Physiol.* **103**, 523–534 (2018).
33. Harada, M., Fuse, A. & Tanaka, Y. Measurement of nitric oxide in the rat cerebral cortex during hypercapnoea. *Neuroreport* **8**, 999–1002 (1997).
34. Harik, S. I., Prado, R., Busto, R. & Ginsberg, M. D. Increased cerebral blood flow during hypercapnia is not affected by lesion of the nucleus *locus coeruleus*. *Stroke* **17**, 1235–1238 (1986).
35. Harris, A. P., Ohata, H. & Koehler, R. C. Role of nitric oxide in cerebrovascular reactivity to NMDA and hypercapnia during prenatal development in sheep. *Int. J. Dev. Neurosci.* **26**, 47–55 (2008).
36. Heinert, G., Casadei, B. & Paterson, D. J. Hypercapnic cerebral blood flow in spontaneously hypertensive rats. *J. Hypertens*. **16**, 1491–1498 (1998).
37. Heinert, G., Nye, P. C. G. & Paterson, D. J. Nitric oxide and prostaglandin pathways interact in the regulation of hypercapnic cerebral vasodilatation. *Acta Physiol. Scand.* **166**, 183–193 (1999).
38. Heinonen, K., Fedinec, A. & Leffler, C. W. Dexamethasone pre-treatment attenuates cerebral vasodilative responses to hypercapnia and augments vasoconstrictive responses to hyperventilation in newborn pigs. *Pediatr. Res*. **53**, 260–265 (2003).
39. Hoffman, W. E., Albrecht, R. F. & Miletich, D. J. The role of adenosine in CBF increases during hypoxia in young vs aged rats. *Stroke* **15**, 124–129 (1984).
40. Hoffman, W. E., Albrecht, R. F., Pelligrino, D. & Miletich, D. J. Effects of prostaglandins on the cerebral circulation in the coat. *Prostaglandins* **23**, 897–905 (1982).
41. Hoiland, R. L. et al. Carbon dioxide-mediated vasomotion of extra-cranial cerebral arteries in humans: a role for prostaglandins? *J. Physiol*. **594**, 3463–3481 (2016).
42. Hoiland, R. L. et al. Indomethacin-induced impairment of regional cerebrovascular reactivity: Implications for respiratory control. *J. Physiol*. **593**, 1291–1306 (2015).
43. Horvath, I., Sandor, N. T., Ruttner, Z. & McLaughlin, A. C. Role of nitric oxide in regulating cerebrocortical oxygen consumption and blood flow during hypercapnia. *J. Cereb. Blood Flow Metab.* **14**, 503–509 (1994).
44. Hosford, P. S. et al. A critical role for the ATP-sensitive potassium channel subunit Kir6.1 in the control of cerebral blood flow. *J. Cereb. Blood Flow Metab*. **39**, 2089–2095 (2019).
45. Howarth, C. et al. A critical role for astrocytes in hypercapnic vasodilation in brain. *J. Neurosci.* **37**, 2403–2414 (2017).
46. Hudetz, A. G., Smith, J. J., Lee, J. G., Bosnjak, Z. J. & Kampine, J. P. Modification of cerebral laser-Doppler flow oscillations by halothane, PCO_2, and nitric oxide synthase blockade. *Am. J. Physiol. - Hear. Circ. Physiol.* **269**, (1995).
47. Iadecola, C. & Xu, X. Nitro-L-arginine attenuates hypercapnic cerebrovasodilation without affecting cerebral metabolism. *Am. J. Physiol*. **266**, R518-25 (1994).
48. Iadecola, C. & Zhang, F. Nitric oxide-dependent and -independent components of cerebrovasodilation elicited by hypercapnia. *Am. J. Physiol. - Regul. Integr. Comp. Physiol*. **266**, (1994).

49. Iadecola, C. & Zhang, F. Permissive and obligatory roles of NO in cerebrovascular responses to hypercapnia and acetylcholine. *Am. J. Physiol.* **271**, R990–R1001 (1996).
50. Iadecola, C., Zhang, F. & Xu, X. Role of nitric oxide synthase-containing vascular nerves in cerebrovasodilation elicited from cerebellum. *Am. J. Physiol. - Regul. Integr. Comp. Physiol*. **264**, (1993).
51. Iadecola, C., Zhang, F. & Xu, X. SIN-1 reverses attenuation of hypercapnic cerebrovasodilation by nitric oxide synthase inhibitors. *Am. J. Physiol. - Regul. Integr. Comp. Physiol*. **267**, (1994).
52. Ide, K., Worthley, M., Anderson, T. & Poulin, M. J. Effects of the nitric oxide synthase inhibitor L-NMMA on cerebrovascular and cardiovascular responses to hypoxia and hypercapnia in humans. *J. Physiol.* **584**, 321–332 (2007).
53. Irikura, K. et al. Cerebrovascular alterations in mice lacking neuronal nitric oxide synthase gene expression. *Proc. Natl. Acad. Sci. U. S. A.* **92**, 6823–7 (1995).
54. Irikura, K., Maynard, K. I., Lee, W. S. & Moskowitz, M. A. L-NNA decreases cortical hyperemia and brain cGMP levels following CO_2 inhalation in Sprague-Dawley rats. *Am. J. Physiol. - Hear. Circ. Physiol*. **267**, (1994).
55. Ito, H., Kinoshita, T., Tamura, Y., Yokoyama, I. & Iida, H. Effect of intravenous dipyridamole on cerebral blood flow in humans: A PET study. *Stroke* **30**, 1616–1620 (1999).
56. Ivancev, V. et al. Effects of indomethacin on cerebrovascular response to hypercapnea and hypocapnea in breath-hold diving and obstructive sleep apnea. *Respir. Physiol. Neurobiol*. **166**, 152–158 (2009).
57. Jackson, E. K. et al. Prostaglandin biosynthesis does not participate in hypercapnia-induced cerebral vasodilatation in the dog. *J. Pharmacol. Exp. Ther.* **226**, 486–492 (1983).
58. Kannurpatti, S. S. & Biswal, B. B. Mitochondrial Ca^{2+} uniporter blockers influence activation-induced CBF response in the rat somatosensory cortex. *J. Cereb. Blood Flow Metab*. **28**, 772–785 (2008).
59. Kastrup, A., Happe, V., Hartmann, C. & Schabet, M. Gender-related effects of indomethacin on cerebrovascular CO_2 reactivity. *J. Neurol. Sci*. **162**, 127–132 (1999).
60. Komjáti, K., Greenberg, J. H., Reivich, M. & Sándor, P. Interactions between the endothelium-derived relaxing factor/nitric oxide system and the endogenous opiate system in the modulation of cerebral and spinal vascular CO_2 responsiveness. *J. Cereb. Blood Flow Metab.* **21**, 937–944 (2001).
61. Kontos, H. A., Wei, E. P., Povlishock, J. T. & Christman, C. W. Oxygen radicals mediate the cerebral arteriolar dilation from arachidonate and bradykinin in cats. *Circ. Res.* **55**, 295–303 (1984).
62. Leffler, C. W. et al. Activated oxygen species do not mediate hypercapnia-induced cerebral vasodilation in newborn pigs. *Am. J. Physiol.* **261**, H335-42 (1991).
63. Leffler, C. W. et al. Effects of indomethacin on cerebral vasodilator responses to arachidonic acid and hypercapnia in newborn pigs. *Pediatr. Res*. **33**, 609–614 (1993).
64. Leffler, C. W., Mirro, R., Pharris, L. J. & Shibata, M. Permissive role of prostacyclin in cerebral vasodilation to hypercapnia in newborn pigs. *Am. J. Physiol.* **267**, (1994).
65. Ma, J. et al. L-NNA-sensitive regional cerebral blood flow augmentation during hypercapnia in type III NOS mutant mice. *Am. J. Physiol*. **271**, H1717-9 (1996).
66. Markus, H. S., Vallance, P. & Brown, M. M. Differential effect of three cyclooxygenase inhibitors on human cerebral blood flow velocity and carbon dioxide reactivity. *Stroke* **25**, 1760–1764 (1994).
67. McCalden, T. A., Nath, R. G. & Thiele, K. The role of prostacyclin in the hypercapnic and hypoxic cerebrovascular dilations. *Life Sci*. **34**, 1801–1807 (1984).
68. McPherson, R. W., Kirsch, J. R., Ghaly, R. F. & Traystman, R. J. Effect of nitric oxide synthase inhibition on the cerebral vascular response to hypercapnia in primates. *Stroke* **26**, 682–7 (1995).
69. Meng, W., Tobin, J. R. & Busija, D. W. Glutamate-induced cerebral vasodilation is mediated by nitric oxide through N-methyl-D-aspartate receptors. *Stroke* **26**, 857–62; discussion 863 (1995).
70. Meno, J. R., Crum, A. V. & Winn, H. R. Effect of adenosine receptor blockade on pial arteriolar dilation during sciatic nerve stimulation. *Am. J. Physiol. - Hear. Circ. Physiol.* **281**, 2018–2027 (2001).
71. Morii, S., Ngai, A. C., Ko, K. R. & Winn, H. R. Role of adenosine in regulation of cerebral blood flow: Effects of theophylline during normoxia and hypoxia. *Am. J. Physiol. - Hear. Circ. Physiol.* **253**, (1987).

72. Nagayama, M., Niwa, K., Nagayama, T., Ross, M. E. & Iadecola, C. The cyclooxygenase-2 inhibitor NS-398 ameliorates ischemic brain injury in wild-type mice but not in mice with deletion of the inducible nitric oxide synthase gene. *J. Cereb. Blood Flow Metab*. **19**, 1213–1219 (1999).
73. Najarian, T. et al. Prolonged hypercapnia-evoked cerebral hyperemia via K+ channel- and prostaglandin E_2-dependent endothelial nitric oxide synthase induction. *Circ. Res*. **87**, 1149–1156 (2000).
74. Niwa, K., Araki, E., Morham, S. G., Ross, M. E. & Iadecola, C. Cyclooxygenase-2 contributes to functional hyperemia in whisker-barrel cortex. *J. Neurosci*. **20**, 763–770 (2000).
75. Niwa, K., Haensel, C., Ross, M. E. & Iadecola, C. Cyclooxygenase-1 participates in selected vasodilator responses of the cerebral circulation. *Circ. Res*. **88**, 600–608 (2001).
76. Niwa, K., Lindauer, U., Villringer, A. & Dirnagl, U. Blockade of nitric oxide synthesis in rats strongly attenuates the CBF response to extracellular acidosis. *J. Cereb. Blood Flow Metab*. **13**, 535–539 (1993).
77. Nnorom, C. C. et al. Contributions of K_{ATP} and K_{Ca} channels to cerebral arteriolar dilation to hypercapnia in neonatal brain. *Physiol. Rep*. **2**, (2014).
78. Okamoto, H., Hudetz, A. G., Roman, R. J., Bosnjak, Z. J. & Kampine, J. P. Neuronal NOS-derived NO plays permissive role in cerebral blood flow response to hypercapnia. *Am. J. Physiol. - Hear. Circ. Physiol*. **272**, (1997).
79. Parfenova, H., Shibata, M., Zuckerman, S. & Leffler, C. W. CO_2 and cerebral circulation in newborn pigs: Cyclic nucleotides and prostanoids in vascular regulation. *Am. J. Physiol. - Hear. Circ. Physiol.* **266**, (1994).
80. Pelligrino, D. A., Koenig, H. M. & Albrecht, R. F. Nitric oxide synthesis and regional cerebral blood flow responses to hypercapnia and hypoxia in the rat. *J. Cereb. Blood Flow Metab.* **13**, 80–87 (1993).
81. Pelligrino, D. A., Wang, Q., Koenig, H. M. & Albrecht, R. F. Role of nitric oxide, adenosine, N-methyl-d-aspartate receptors, and neuronal activation in hypoxia-induced pial arteriolar dilation in rats. *Brain Res*. **704**, 61–70 (1995).
82. Peltonen, G. L. et al. Cerebral blood flow regulation in women across menstrual phase: Differential contribution of cyclooxygenase to basal, hypoxic, and hypercapnic vascular tone. *Am. J. Physiol. - Regul. Integr. Comp. Physiol.* **311**, R222–R231 (2016).
83. Peltonen, G. L. et al. Cerebrovascular regulation in men and women: Stimulus-specific role of cyclooxygenase. *Physiol. Rep*. **3**, 1–11 (2015).
84. Phillis, J. W. & DeLong, R. E. An involvement of adenosine in cerebral blood flow regulation during hypercapnia. *Gen. Pharmacol.* **18**, 133–139 (1987).
85. Phillis, J. W. & O'Regan, M. H. Effects of adenosine receptor antagonists on pial arteriolar dilation during carbon dioxide inhalation. *Eur. J. Pharmacol.* **476**, 211–219 (2003).
86. Phillis, J. W., Lungu, C. L., Barbu, D. E. & O'Regan, M. H. Adenosine's role in hypercapnia-evoked cerebral vasodilation in the rat. *Neurosci. Lett*. **365**, 6–9 (2004).
87. Pickard, J. D. & Mackenzie, E. T. Inhibition of prostaglandin synthesis and the response of baboon cerebral circulation to carbon dioxide. *Nat. New Biol.* **245**, 187–8 (1973).
88. Pickard, J., Tamura, A., Stewart, M., McGeorge, A. & Fitch, W. Prostacyclin, indomethacin and the cerebral circulation. *Brain Res.* **197**, 425–431 (1980).
89. Pickles, H. et al. Effect of indomethacin on cerebral blood flow, carbon dioxide reactivity and the response to epoprostenol (prostacyclin) infusion in man. *J. Neurol. Neurosurg. Psychiatry* **47**, 51–55 (1984).
90. Pourcyrous, M. et al. Cerebrovascular responses to therapeutic dose of indomethacin in newborn pigs. *Pediatr. Res*. **45**, 582–7 (1999).
91. Quintana, A., Raczka, E. & Quintana, M. A. Effects of indomethacin and diclofenac on cerebral blood flow in hypercapnic conscious rats. *Eur. J. Pharmacol*. **149**, 385–388 (1988).
92. Reid, J. M., Davies, A. G., Ashcroft, F. M. & Paterson, D. J. Effect of L-NMMA, cromakalim, and glibenclamide on cerebral blood flow in hypercapnia and hypoxia. *Am. J. Physiol. - Hear. Circ. Physiol.* **269**, (1995).
93. Reid, J. M., Paterson, D. J., Ashcroft, F. M. & Bergel, D. H. The effect of tolbutamide on cerebral blood flow during hypoxia and hypercapnia in the anaesthetized rat. *Pflügers Arch. Eur. J. Physiol.* **425**, 362–364 (1993).

94. Rosenblum, W. I., Wei, E. P. & Kontos, H. A. Dilation of rat brain arterioles by hypercapnia in vivo can occur even after blockade of guanylate cyclase by ODQ. *Eur. J. Pharmacol.* **448**, 201–206 (2002).
95. Sakabe, T. & Siesjö, B. K. The effect of indomethacin on the blood flow-metabolism couple in the brain under normal, hypercapnic and hypoxic conditions. *Acta Physiol. Scand.* **107**, 283–84 (1979).
96. Sandor, P., Komjati, K., Reivich, M. & Nyary, I. Major role of nitric oxide in the mediation of regional CO_2 responsiveness of the cerebral and spinal cord vessels of the cat. *J. Cereb. Blood Flow Metab*. **14**, 49–58 (1994).
97. Schmetterer, L. et al. Role of NO in the O_2 and CO_2 responsiveness of cerebral and ocular circulation in humans. *Am. J. Physiol. - Regul. Integr. Comp. Physiol*. **273**, (1997).
98. Scremin, O. U., Sonnenschein, R. R. & Rubinstein, E. H. Cholinergic cerebral vasodilatation in the rabbit: Absence of concomitant metabolic activation. *J. Cereb. Blood Flow Metab.* **2**, 241–247 (1982).
99. Shoemaker, L. N. et al. Indomethacin markedly blunts cerebral perfusion and reactivity, with little cognitive consequence in healthy young and older adults. *J. Physiol.* **599**, 1097–1113 (2021).
100. Simpson, R. E. & Phillis, J. W. Adenosine deaminase reduces hypoxic and hypercapnic dilatation of rat pial arterioles: evidence for mediation by adenosine. *Brain Res*. **553**, 305–308 (1991).
101. Simpson, R. E., Phillis, J. W. & Buchanan, J. Nifedipine reduces the increases in cerebral blood flow during hypercapnic episodes. *Gen. Pharmacol.* **22**, 359–364 (1991).
102. Smith, J. J. et al. The role of nitric oxide in the cerebrovascular response to hypercapnia. *Anesth. Analg*. **84**, 363–369 (1997).
103. St. Lawrence, K. S. et al. Effects of indomethacin on cerebral blood flow at rest and during hypercapnia: An arterial spin tagging study in humans. *J. Magn. Reson. Imaging* **15**, 628–635 (2002).
104. Takenaka, M., Iida, H., Iida, M., Uchida, M. & Dohi, S. The comparative effects of prostaglandin E_1 and nicardipine on cerebral microcirculation in rabbits. *Anesth. Analg.* **96**, 1139–1144 (2003).
105. Thompson, B. G., Pluta, R. M., Girton, M. E. & Oldfield, E. H. Nitric oxide mediation of chemoregulation but not autoregulation of cerebral blood flow in primates. *J. Neurosurg.* **84**, 71–78 (1996).
106. Uekawa, K. et al. Obligatory role of EP1 receptors in the increase in cerebral blood flow produced by hypercapnia in the mice. *PLoS One* **11**, 1–16 (2016).
107. Vuletic, V., Drenjancevic, I., Rahelic, D. & Demarin, V. Effect of indomethacin on cerebrovascular reactivity in patients with type 2 diabetes mellitus. *Diabetes Res. Clin. Pract.* **101**, 81–87 (2013).
108. Wagerle, L. C. & Mishra, O. P. Mechanism of CO_2 response in cerebral arteries of the newborn pig: Role of phospholipase, cyclooxygenase, and lipoxygenase pathways. *Circ. Res.* **62**, 1019–1026 (1988).
109. Wang, Q., Bryan, R. M. & Pelligrino, D. A. Calcium-dependent and ATP-sensitive potassium channels and the 'permissive' function of cyclic GMP in hypercapnia-induced pial arteriolar relaxation. *Brain Res.* **793**, 187–196 (1998).
110. Wang, Q., Bryowsky, J., Minshall, R. D. & Pelligrino, D. A. Possible obligatory functions of cyclic nucleotides in hypercapnia- induced cerebral vasodilation in adult rats. *Am. J. Physiol. - Hear. Circ. Physiol*. **276**, (1999).
111. Wang, Q., Paulson, O. B. & Lassen, N. A. Effect of nitric oxide blockade by N^G-nitro-l-arginine on cerebral blood flow response to changes in carbon dioxide tension. *J. Cereb. Blood Flow Metab*. **12**, 947–953 (1992).
112. Wang, Q., Paulson, O. B. & Lassen, N. A. Indomethacin abolishes cerebral blood flow increase in response to acetazolamide-induced extracellular acidosis: A mechanism for its effect on hypercapnia? *J. Cereb. Blood Flow Metab.* **13**, 724–727 (1993).
113. Wang, Q., Pelligrino, D. A., Baughman, V. L., Koenig, H. M. & Albrecht, R. F. The role of neuronal nitric oxide synthase in regulation of cerebral blood flow in normocapnia and hypercapnia in rats. *J. Cereb. Blood Flow Metab*. **15**, 774–778 (1995).
114. Wang, Q., Pelligrino, D. A., Koenig, H. M. & Albrecht, R. F. The role of endothelium and nitric oxide in rat pial arteriolar dilatory responses to CO_2 in vivo. *J. Cereb. Blood Flow Metab.* **14**, 944–951 (1994).
115. Wang, Q., Pelligrino, D. A., Paulson, O. B. & Lassen, N. A. Comparison of the effects of N^G-nitro-L-arginine and indomethacin on the hypercapnic cerebral blood flow increase in rats. *Brain Res*. **641**, 257–264 (1994).

116. Wei, E. P., Ellis, E. F. & Kontos, H. A. Role of prostaglandins in pial arteriolar response to CO_2 and hypoxia. *Am. J. Physiol. - Hear. Circ. Physiol.* **7**, (1980).
117. Wennmalm, A. et al. Central and peripheral haemodynamic effects of non-steroidal anti-inflammatory drugs in man. *Arch. Toxicol. Suppl.* **7**, 350–9 (1984).
118. Wennmalm, Å., Eriksson, S. & Wahren, J. Effect of indomethacin on basal and carbon dioxide stimulated cerebral blood flow in man. *Clin. Physiol*. **1**, 227–234 (1981).
119. White, R. P., Deane, C., Vallance, P. & Markus, H. S. Nitric oxide synthase inhibition in humans reduces cerebral blood flow but not the hyperemic response to hypercapnia. *Stroke* **29**, 467–472 (1998).
120. Williams, J. L., Jones, S. C., Page, R. B. & Bryan, R. M. Vascular responses of choroid plexus during hypercapnia in rats. *Am. J. Physiol. - Regul. Integr. Comp. Physiol*. **260**, (1991).
121. Willis, A. P. & Leffler, C. W. Endothelial NO and prostanoid involvement in newborn and juvenile pig pial arteriolar vasomotor responses. *Am. J. Physiol. - Hear. Circ. Physiol.* **281**, 2366–2377 (2001).
122. Wołk, R., Nowicki, D., Siemińska, J. & Trzebski, A. Role of the endogenous nitric oxide in the vasodilatory tone and CO_2 responsiveness of the rostral ventrolateral medulla microcirculation in the rat. *J. Physiol. Pharmacol.* **46**, 127–39 (1995).
123. Wolk, R., Sieminska, J. & Trzebski, A. Dual response of cerebrocortical blood flow and arterial blood pressure to transient CO_2 stimulus after inhibition of nitric oxide synthesis in rats. *Acta Neurobiol. Exp. (Wars).* **55**, 73–84 (1995).
124. Xie, A. et al. Influence of cerebral blood flow on breathing stability. *J. Appl. Physiol.* **106**, 850–856 (2009).
125. Xie, A. et al. Influence of cerebrovascular function on the hypercapnic ventilatory response in healthy humans. *J. Physiol*. **577**, 319–329 (2006).
126. Xu, H. L., Koenig, H. M., Ye, S., Feinstein, D. L. & Pelligrino, D. Influence of the glia limitans on pial arteriolar relaxation in the rat. *Am. J. Physiol. Heart Circ. Physiol*. **287**, H331–H339 (2004).
127. Yang, G., Zhang, Y., Ross, M. E. & Iadecola, C. Attenuation of activity-induced increases in cerebellar blood flow in mice lacking neuronal nitric oxide synthase. *Am. J. Physiol. Heart Circ. Physiol*. **285**, 298–304 (2003).
128. Zhang, F., Slungaard, A., Gregory, M., Vercellotti & Iadecola, C. Superoxide-dependent cerebrovascular effects of homocysteine. *Am. J. Physiol. - Regul. Integr. Comp. Physiol*. **274**, (1998).
129. Zhang, F., Xu, S. & Iadecola, C. Role of nitric oxide and acetylcholine in neocortical hyperemia elicited by basal forebrain stimulation: evidence for an involvement of endothelial nitric oxide. *Neuroscience* **69**, 1195–204 (1995).
130. Zhang, Y. & Leffler, C. W. Compensatory role of NO in cerebral circulation of piglets chronically treated with indomethacin. *Am. J. Physiol. - Regul. Integr. Comp. Physiol*. **282**, 400–410 (2002).
131. Zuckerman, S. L., Armstead, W. M., Hsu, P., Shibata, M. & Leffler, C. W. Age dependence of cerebrovascular response mechanisms in domestic pigs. *Am. J. Physiol. Heart Circ. Physiol.* **271**, 8–10 (1996).
