# Supplementary material for: CO2 signaling mediates neurovascular coupling in the cerebral cortex
Source: Nat Commun. 2022 Apr 19;13:2125. doi: 10.1038/s41467-022-29622-9 (PMC9019094; doi:10.1038/s41467-022-29622-9)
Supplement: Supplementary file 3 — Reporting Summary [file 41467_2022_29622_MOESM3_ESM.pdf]

nature research

Corresponding author(s): Alexander V. Gourine

Last updated by author(s): February 11, 2022

Reporting Summary

Nature Research wishes to improve the reproducibility of the work that we publish. This form provides structure for consistency and transparency in reporting. For further information on Nature Research policies, see our Editorial Policies and the Editorial Policy Checklist.

Statistics

For all statistical analyses, confirm that the following items are present in the figure legend, table legend, main text, or Methods section.

n/a	Confirmed	
☐	☒	The exact sample size (*n*) for each experimental group/condition, given as a discrete number and unit of measurement
☐	☒	A statement on whether measurements were taken from distinct samples or whether the same sample was measured repeatedly
☐	☒	The statistical test(s) used AND whether they are one- or two-sided<br>*Only common tests should be described solely by name; describe more complex techniques in the Methods section.*
☒	☐	A description of all covariates tested
☐	☒	A description of any assumptions or corrections, such as tests of normality and adjustment for multiple comparisons
☐	☒	A full description of the statistical parameters including central tendency (e.g. means) or other basic estimates (e.g. regression coefficient) AND variation (e.g. standard deviation) or associated estimates of uncertainty (e.g. confidence intervals)
☐	☒	For null hypothesis testing, the test statistic (e.g. *F*, *t*, *r*) with confidence intervals, effect sizes, degrees of freedom and *P* value noted<br>*Give P values as exact values whenever suitable.*
☒	☐	For Bayesian analysis, information on the choice of priors and Markov chain Monte Carlo settings
☒	☐	For hierarchical and complex designs, identification of the appropriate level for tests and full reporting of outcomes
☒	☐	Estimates of effect sizes (e.g. Cohen's *d*, Pearson's *r*), indicating how they were calculated

Our web collection on statistics for biologists contains articles on many of the points above.

Software and code

Policy information about availability of computer code

Data collection	Power 1401, Spike2-Version7
Data analysis	Spike2-Version7; GraphPad-Prism-Version8;

For manuscripts utilizing custom algorithms or software that are central to the research but not yet described in published literature, software must be made available to editors and reviewers. We strongly encourage code deposition in a community repository (e.g. GitHub). See the Nature Research guidelines for submitting code & software for further information.

Data

Policy information about availability of data

All manuscripts must include a data availability statement. This statement should provide the following information, where applicable:

- Accession codes, unique identifiers, or web links for publicly available datasets
- A list of figures that have associated raw data
- A description of any restrictions on data availability

The data that support the findings of this study are presented as Supplementary Information, within the Source Data File and available from the corresponding authors upon request. The source data underlying Figures 1; 2c,e-g; 3c-f; 4d-g; and Supplementary Figures 1c,d; 2b,c; 3a; 4b,c; 5a-d; 6b are provided as a Source Data file.

Field-specific reporting

Please select the one below that is the best fit for your research. If you are not sure, read the appropriate sections before making your selection.

- [x] Life sciences
- [ ] Behavioural & social sciences
- [ ] Ecological, evolutionary & environmental sciences

For a reference copy of the document with all sections, see nature.com/documents/nr-reporting-summary-flat.pdf

Life sciences study design

All studies must disclose on these points even when the disclosure is negative.

Sample size	The paper describes the results of the experiments performed using in vivo animal models. Power calculations for the in vivo animal studies are described below. The significance was set at 0.05 and the beta was set at 0.20. From years of relevant research experience, we expect to detect a significant difference with a minimum of 5 animals per experimental group, if the treatments cause differences between means that are as large as 2.25 standard deviations (SD), likely to be a physiologically significant difference. If the difference is as small as 1.75 SD, we increase sample sizes to 9. Although, increasing the sample sizes per group to 15 may enable to detect a difference between means of 1.25 SD, it is doubtful these differences would have a biological significance.
Data exclusions	No data was excluded from the analysis.
Replication	All experiments were independently conducted at least 5 times in the in vivo studies. All attempts to replicate the findings were consistently successful in the present study.
Randomization	Randomization is not relevant to this study, since experimental subjects were obtained from a genetically homogeneous colony and then assigned to different experimental groups according to the treatment.
Blinding	All experimenters were blinded to the condition or treatment/genotype of experimental subjects and to the identity of the experimental groups.

Reporting for specific materials, systems and methods

We require information from authors about some types of materials, experimental systems and methods used in many studies. Here, indicate whether each material, system or method listed is relevant to your study. If you are not sure if a list item applies to your research, read the appropriate section before selecting a response.

Materials & experimental systems

n/a	Involved in the study	
[ ]	[x]	Antibodies
[x]	[ ]	Eukaryotic cell lines
[x]	[ ]	Palaeontology and archaeology
[ ]	[x]	Animals and other organisms
[x]	[ ]	Human research participants
[x]	[ ]	Clinical data
[x]	[ ]	Dual use research of concern

Methods

n/a	Involved in the study	
[x]	[ ]	ChIP-seq
[x]	[ ]	Flow cytometry
[ ]	[x]	MRI-based neuroimaging

Antibodies

Antibodies used	Primary mouse monoclonal anti-NBCe1 (Santa Cruz Biotechnology, Cat # sc-515543, Lot # I2216); secondary anti-mouse antibody Dylight 488 (ThermoFisher, Cat #35502).
Validation	The antibodies were validated using negative control protocols and using cell/tissue samples of specific gene knockout animals. We employ these antibodies routinely and we have published research articles demonstrating their specificity. In the present study immunohistochemistry was used to show that tamoxifen- induced recombination is taking place specifically in astrocytes of NBCe1 flox/flox:GLAST CreERT2/+ mice. The specificity of anti-NBCe1 antibody was confirmed by observing lack of immunostaining in cultured astrocytes of global NBCe1 knockout mice. The validation data are available from the corresponding author upon request.

Animals and other organisms

Policy information about studies involving animals; ARRIVE guidelines recommended for reporting animal research

Laboratory animals	Young adult laboratory male Sprague-Dawley rats (2-3 mo old); young adult male NBCe1 knockout and control mice on C57Bl/6J background (3-4 mo old).

Wild animals	The study did not involve any wild animals.
Field-collected samples	The study did not involve any field collected samples.
Ethics oversight	All animal experimentations were performed in accordance with the European Commission Directive 2010/63/EU (European Convention for the Protection of Vertebrate Animals used for Experimental and Other Scientific Purposes) and the UK Home Office (Scientific Procedures) Act (1986) with project approval from the Institutional Animal Care and Use Committees of the University College London and Centro de Estudios Científicos.

Note that full information on the approval of the study protocol must also be provided in the manuscript.

Magnetic resonance imaging

Experimental design

Design type	Task based
Design specifications	For each condition (e.g 5% C02, or 10% inspired CO2), there was a baseline period of 1 minute and a stimulus duration of 1 minute which was repeated three times.
Behavioral performance measures	NA

Acquisition

Imaging type(s)	Arterial Spin Labelling, T2* weighted.
Field strength	9.4T
Sequence & imaging parameters	Single shot gradient echo EPI readout, TR=5000 ms, inflow time (TI)=2000 ms, matrix size=64×64, FOV=35 mm × 35mm, TE=10 ms, single slice (slice thickness=2mm)
Area of acquisition	Single coronal slice centered on the forepaw region of the somatosensory cortex (manually positioned based on the anatomical reference scan images)

Diffusion MRI ☐ Used ☒ Not used

Preprocessing

Preprocessing software	NA
Normalization	NA
Normalization template	NA
Noise and artifact removal	In order to partially correct the ASL (ΔM) signal for marked BOLD T2* weighted signal changes that occur when the forepaw stimulus begins/ceases at a time between the labelled and control acquisitions, the labelled image directly after the stimulus begins was substituted by the previous labelled image (captured during the baseline period). Similarly the control image directly after cessation of the forepaw stimulus was substituted by the previous control image (captured during the stimulation period). For some datasets where clear hardware-related instability was present, a de-spiking algorithm was applied to the ASL/BOLD timecourse data.
Volume censoring	NA

Statistical modeling & inference

Model type and settings	For visualization purposes, fixed effects modelling was used to generate activation maps across conditions for individual subjects (using the BOLD signal time-courses)
Effect(s) tested	To generate activation maps for individual animals, we tested for significant increases in the BOLD signal in response to the forepaw stimulation.

Specify type of analysis: ☐ Whole brain ☐ ROI-based ☒ Both

Anatomical location(s)	CBF and BOLD time-series data were extracted from a manually drawn ROI in the unilateral forepaw region of the somatosensory cortex. The ROI was drawn on the baseline functional data based on a map of the mean CBF during activation minus the mean CBF during the interstimulus interval. The ROI was then fixed for all the subsequent conditions.
Statistic type for inference (See Eklund et al. 2016)	Cluster-wise (minimum of 5 voxels)
Correction	FWE

Models & analysis

n/a	Involved in the study
☒	☐ Functional and/or effective connectivity
☒	☐ Graph analysis
☒	☐ Multivariate modeling or predictive analysis
